# Cognitively-Based Compassion Training versus cancer health education to improve health-related quality of life in survivors of solid tumor cancers and their informal caregivers: study protocol for a randomized controlled pilot trial

**DOI:** 10.1186/s13063-019-3320-9

**Published:** 2019-04-29

**Authors:** Thaddeus W. W. Pace, Sally E. Dodds, Alla Sikorskii, Terry A. Badger, Chris Segrin, Lobsang Tenzin Negi, Timothy Harrison, Tracy E. Crane

**Affiliations:** 1 Division of Community and Systems Health Science, College of Nursing, University of Arizona, 1305 N Martin Ave, Tucson, AZ 85721 USA; 2 Department of Psychiatry, College of Medicine, University of Arizona, Tucson, AZ USA; 3 Department of Psychology, College of Science, University of Arizona, Tucson, AZ USA; 4 University of Arizona Cancer Center, Tucson, AZ USA; 5 Department of Psychiatry, College of Osteopathic Medicine, Michigan State University, East Lansing, MI USA; 6 Department of Communication, College of Social and Behavioral Sciences, University of Arizona, Tucson, AZ USA; 7 Emory-Tibet Partnership, Department of Religion, Emory College, Emory University, Atlanta, GA USA; 8 Emory-Tibet Partnership, CBCT Teacher Training, Emory University, Atlanta, GA USA; 9 Division of Biobehavioral Healthscience, College of Nursing, University of Arizona, Tucson, AZ USA

**Keywords:** Cancer survivorship, Health-related quality of life, Compassion meditation, Dyadic interdependence, Inflammation, Cortisol, Active control

## Abstract

**Background:**

Cancer survivors and their informal caregivers (family members, close friends) often experience significant impairments in health-related quality of life (HRQOL), including disruptions in psychological, physical, social, and spiritual well-being both during and after primary cancer treatment. The purpose of this in-progress pilot trial is to determine acceptability and preliminary efficacy (as reflected by effect sizes) of CBCT® (Cognitively-Based Compassion Training) compared with a cancer health education (CHE) attention control to improve the primary outcome of depressive symptoms and secondary outcomes of other HRQOL domains (e.g., anxiety, fatigue), biomarkers of inflammation and diurnal cortisol rhythm, and healthcare utilization-related outcomes in both cancer survivors and informal caregivers.

**Methods:**

Forty dyads consisting of solid tumor survivors who have completed primary treatments (chemotherapy, radiation, surgery) and their informal caregivers, with at least one dyad member with ≥ mild depressive symptoms or anxiety, will be recruited from Tucson, Arizona, USA. Survivor-caregiver dyads will be randomized together to complete either CBCT or CHE. CBCT is a manualized, 8-week, group meditation-based intervention that starts with attention and mindfulness and builds to contemplative practices aimed at cultivating compassion to the self and others. The goal of CBCT is to challenge unexamined assumptions about feelings and behaviors, with a focus on generating spontaneous self-compassion and increased empathic responsiveness and compassion for others. CHE is an 8-week, manualized group intervention that provides cancer-specific education on various topics (e.g., cancer advocacy, survivorship wellness). Patient-reported HRQOL outcomes will be assessed before, immediately after (week 9), and 1 month after CBCT or CHE (week 13). At the same time points, stress-related biomarkers of inflammation (e.g., plasma interleukin-6) and saliva cortisol relevant for survivor and informal caregiver wellness and healthcare utilization will be measured.

**Discussion:**

If CBCT shows acceptability, a larger trial will be warranted and appropriately powered to formally test the efficacy of this dyadic intervention. Interventions such as CBCT directed toward both survivors and caregivers may eventually fill a gap in supportive oncology care programs to improve HRQOL and healthcare utilization in both dyad members.

**Trial registration:**

Clinicaltrials.gov, NCT03459781. Prospectively registered on 9 March 2018.

**Electronic supplementary material:**

The online version of this article (10.1186/s13063-019-3320-9) contains supplementary material, which is available to authorized users.

## Background

Advancements in early detection and treatments have dramatically improved prognosis for the majority of patients diagnosed with solid tumor cancers. Recent trend data show that 5-year survival rates are improving for those treated for colorectal (66%), prostate (99%), and breast cancer (women, 90%) [1]. Despite these gains, health-related quality of life (HRQOL) impairments remain significant for cancer survivors both during and after treatment. For example, after diagnosis and the end of primary treatment, survivors of colorectal, prostate, and breast cancer are likely to experience elevated depressive symptoms (i.e., 28% [2], 18% [3], and 10–25% [4, 5], respectively), fatigue (23–73% [2, 6], 12–21% [7], and 30–80% [8–10], respectively), and anxiety symptoms (including fear of recurrence) [11–16].

While the focus on HRQOL in cancer survivorship is often on cancer survivors themselves, informal caregivers (hereafter referred to as caregivers; i.e., family members and friends who provide supportive care for cancer survivors) also suffer HRQOL impairments. A sizable portion of these caregivers experience increased depressive symptoms and distress, not just around the time of diagnosis and during primary treatments, but also after primary cancer treatments have ended for the cancer survivor [17–19]. Impairments experienced by caregivers are significant for several reasons. Caregivers provide significant supportive care (as much as 60%) to their loved one diagnosed with and treated for cancer [19–21], and HRQOL impairments can undermine their effectiveness in this role. Quality of life of caregivers is important and significant in its own right, and stress experienced by caregivers may have an impact on stress-responsive physiology, including cortisol and inflammation. These stress biological changes may accompany an increased risk for chronic medical and psychiatric illness experienced by caregivers [22–24].

Although there are many interventions to improve HRQOL for cancer survivors, there are few for caregivers, and still fewer that engage both survivors and caregivers together. The dearth of programs that engage survivors and caregivers together is a significant gap. Work by our group has shown that increased depressive symptoms, anxiety, symptom distress (including fatigue), and positive affect that breast (or prostate) cancer survivors experience are interdependent with the same impairments that their caregivers experience, and vice-versa [25, 26]. Caregivers are therefore much more than a “social backdrop” to cancer and its treatment [25]. Instead, HRQOL is shared by both members of a cancer survivor dyad, perhaps by way of “emotional contagion,” or through the multilevel phenomenon whereby stimuli from one individual results in a complimentary emotional or behavioral state in another individual (i.e., the “common fate” model) [27]. We believe that our findings suggest that interventions to promote HRQOL in either cancer survivors or their caregivers should be directed at both members of the dyad simultaneously to leverage emotional contagion to promote the sharing of positive affect, potentially dismantling the possibility of shared negative affect.

CBCT® (Cognitively-Based Compassion Training) is a manualized 8-week, meditation-based intervention that cultivates empathy and prosocial capacity and enhances perceptions of social connection and positive emotions for others [28]. CBCT is “cognitively-based” in that it relies on an analytical method of meditation to gain deeper understanding of a particular topic [29]. The intervention begins by teaching mindfulness practices and builds on this heightened awareness to facilitate constructive emotional states, self-compassion, and feelings of social connectivity [28]. We have found that CBCT improves a number of different HRQOL dimensions in breast cancer survivors, including symptoms of depression, fear of cancer recurrence, and vitality [30]. We have also found that CBCT has other health-related benefits in nonsurvivors, including reduced stress-related inflammation in young adults and foster care adolescents [30–36]. These findings suggest that CBCT is likely to be feasible for use in survivors of other solid tumor cancers and may promote positive changes in key aspects of HRQOL in the same individuals. However, CBCT has not been tested before with survivors other than those of breast cancer who have different treatments and prognoses, or with caregivers of solid tumor cancer survivors. Therefore, in the present pilot trial we will investigate acceptability of CBCT, as well as an active attention control condition called cancer health education (CHE) for both solid tumor cancer survivors and their caregivers.

Besides acceptability of CBCT and CHE, we will also evaluate effect sizes for the differences between CBCT and CHE on several outcomes especially relevant for survivors and caregivers. The primary outcome among these will be depressive symptoms, a key domain of psychological HRQOL. Secondary outcomes will include other HRQOL domains (anxiety, positive affect, fatigue, empathy, feelings of social connection), global HRQOL, dyadic function, and self-compassion. We will also assess stress-related biomarkers and healthcare utilization as secondary outcomes. Considerable evidence, including work by our group, suggests that HRQOL impairments in cancer survivors are causally linked to changes in stress-related physiological function, including diurnal cortisol production and biomarkers of inflammation (plasma interleukin (IL)-1β, IL-6, and tumor necrosis factor (TNF)-α; peripheral blood mononuclear cell (PBMC) nuclear factor-κB (NF-κB)) [37, 38]. HRQOL impairments (e.g., depression) have also been found to be associated with poor use of healthcare resources in cancer survivors [39]. The current trial will begin to extend our earlier findings with breast cancer survivors to survivors of other cancers, and CBCT will be conducted for the first time in a form where both cancer survivors and their caregivers take part in CBCT together. If CBCT and CHE are acceptable to survivors and caregivers, a larger trial in the future will be justified. To avoid the possibility of a floor effect with the outcomes assessed (especially psychological HRQOL; i.e., depression and anxiety), and because prior research with CBCT suggests brain systems implicated in compassion may show beneficial change when depression symptoms improve while learning CBCT [34], the study will enroll dyads with a survivor and/or caregiver with ≥ mild depressive and/or anxiety symptoms (according to PROMIS distress-depression or distress-anxiety four-item scales).

The overarching prediction of the current pilot trial is that participation in CBCT or CHE will be acceptable to solid tumor cancer survivors and their caregivers. We will also capture effect sizes for outcomes noted above for the comparisons of participants randomized to either CBCT or CHE. The following aims guide the pilot trial.

### Study aims and hypotheses

Aim 1 is to demonstrate acceptability of CBCT and CHE for solid tumor cancer survivors and their caregivers. We predict that both CBCT and CHE will be acceptable for use with survivor-caregiver dyads, as reflected by > 75% average attendance at weekly CBCT classes, and > 75% average attendance at weekly CHE classes.

Aim 2 is to estimate effect sizes for the differences between CBCT and CHE at weeks 9 and 13 on HRQOL-related outcomes including outcomes of psychological HRQOL (depressive symptoms (primary outcome), anxiety, positive affect, and self-compassion), physical HRQOL (fatigue), and social HRQOL (empathy, feelings of social connection/isolation, dyadic function) as well as global well-being. Differences between groups at weeks 9 and 13 on HRQOL-related outcomes will be used to inform sample size calculations for a full-scale clinical trial.

Aim 3 is to estimate effect sizes between groups at weeks 9 and 13 on stress-related biomarkers of inflammation (plasma IL-6, IL-1β, TNF-α, NF-κB pathway activation), as well as diurnal saliva cortisol rhythm in survivor-caregiver dyads randomized to CBCT compared with survivor-caregiver dyads randomized to CHE. Differences between groups at weeks 9 and 13 on biomarker outcomes will be used to inform sample size calculations for a full-scale clinical trial.

Aim 4 is to estimate effect sizes for the differences between CBCT and CHE at weeks 9 and 13 on healthcare utilization (i.e., keeping appointments, use of preventive services, hospitalizations, and use of urgent care or emergency department services), and patient activation (i.e., motivation, knowledge, skills and confidence in managing personal health). Differences between groups at weeks 9 and 13 on healthcare utilization outcomes will be used to inform sample size calculations for a full-scale clinical trial.

## Methods

### Study design, allocation procedures, and blinding

**Fig. 1:**
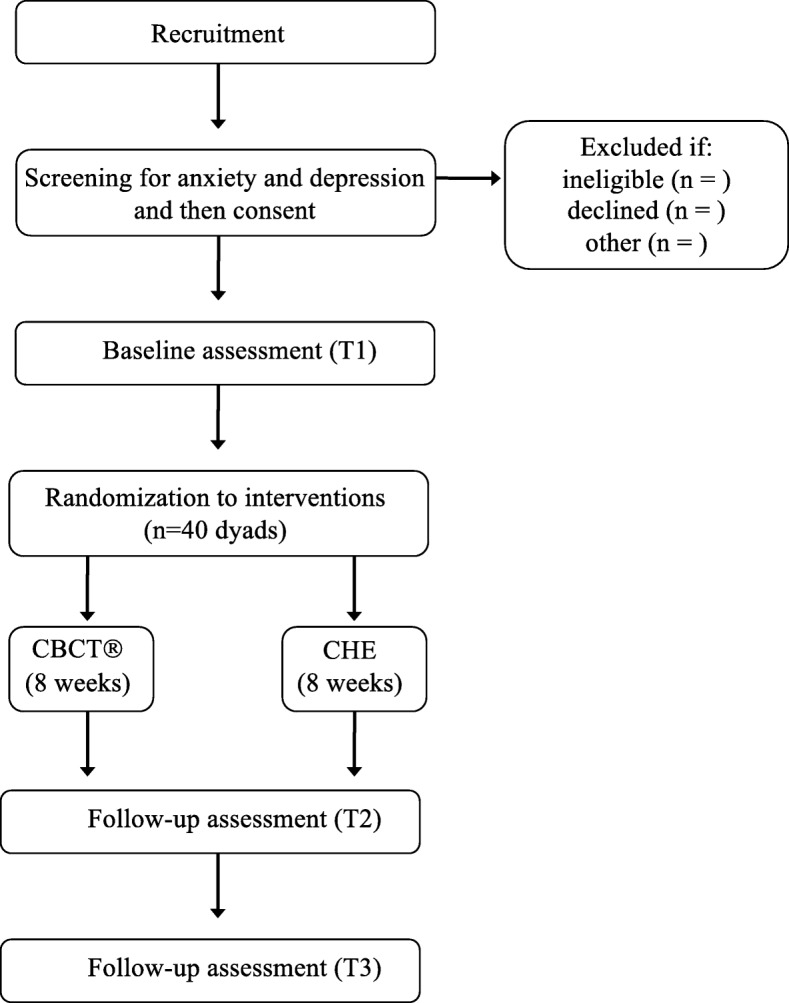
Diagram of study flow. CBCT® Cognitively-Based Compassion Training, CHE cancer health education

This interventional study uses a randomized controlled trial design to compare CBCT with CHE to determine effect sizes in study outcomes (see below). Participants review and sign the informed consent form in the presence of either the Principal Investigator or a study coordinator after a script is read aloud to them that describes the study. Enrolled dyads are randomized 1:1 in blocks of two dyads to either CBCT (*n* = 20 dyads) or the attention control (*n* = 20 dyads). CBCT and CHE groups are run concurrently. Group assignment to either CBCT or CHE is revealed after the baseline (T1) assessment is complete. This study protocol follows the Standard Protocol Items: Recommendations for Interventional Trials (SPIRIT) guidelines (see the SPIRIT checklist for this protocol in Additional file 1). Enrollment began in March 2018, and is recruiting at the time this report was prepared. We are running 3–4 cohorts of CBCT and 3–4 cohorts of the CHE group over 24 months, with 4–10 dyads in each cohort. HRQOL and healthcare adherence assessments as well as collection of biomarker samples are conducted before (T1), immediately after either 8-week intervention (T2), and then again 4–5 weeks later (T3). Overall study flow is depicted in Fig. 1. A study coordinator schedules assessment visits at the University of Arizona College of Nursing. Participants are also contacted the day before each assessment visit for a reminder, and to convey information about finding their way to the assessment site. If participants fail to complete study assessments at T2 (week 9), we attempt to collect data from them at T3 (week 13). Class attendance and at-home practice time (CBCT group only) are collected as measures of intervention engagement.

The randomization (allocation) sequence used to assign dyads to CBCT or CHE is generated using a computer program by someone not involved in other aspects of the study procedures (see acknowledgements below). Dyads are informed of their group assignment by a study coordinator. The coordinator who informs participants about their group assignment is not involved in recruitment, scheduling of study assessments, or administration of study assessments. The same coordinator only learns about a particular dyad’s group assignment from the individual who generates the allocation sequence after that dyad has completed T1 assessments. All other study personnel who are not interventionists remain blinded to group assignment throughout the study. Participants are asked to not discuss their group assignment with anyone other than a study coordinator who is not involved in administration of study assessments. In the event of an adverse event that is either related or unrelated to the trial unblinding will be permitted so that other members of the study team (i.e., the Principal Investigator or clinician co-Investigators) are able to respond appropriately. Participation in the study may be stopped if a participant is unable to complete a study assessment per protocol because of health status changes.

### Research ethics approval

All procedures have been reviewed and approved by the University of Arizona Institutional Review Board (IRB) and have been found to be acceptable according to relevant policies of the University of Arizona as well as state and United States federal regulations designed to protect the rights and welfare of research participants. Any changes made to the study protocol will be approved by the IRB as a protocol amendment and communicated to all members of the study team.

### Participants

This study is enrolling survivors of solid tumor cancers who have completed cancer treatment (except for hormonal therapies) and their caregivers. For the current study, caregivers are defined as an individual (one caregiver per survivor) identified by the survivor as either a family member or close friend (i.e., fictive kin) who has provided and/or continues to provide support to the survivor. Survivors and caregivers can be either gender, and any race/ethnicity.

To be included in the study, cancer survivors and caregivers must be: 1) aged 21 or older; 2) cognitively oriented in time, place, and person; 3) able to speak and understand English; and 4) able to travel to a centralized location to attend CBCT or CHE group sessions. Inclusion criteria specific to cancer survivors are that they must have: 1) a solid tumor cancer diagnosis; and 2) have completed cancer treatment (surgery, radiation, chemotherapy) except for hormonal therapies (e.g., aromatase inhibitors, androgen suppression therapy) a minimum of 3 months and a maximum of 10 years before starting CBCT or CHE. An inclusion criterion specific to caregivers is that they must be named by their cancer survivor. Cancer survivor and caregivers are excluded from the study if they: 1) have a self-reported diagnosis of a major mental illness (e.g., psychosis or uncontrolled major depression); 2) are a nursing home resident; and 3) have an ongoing compassion meditation practice (as determined by the Principal Investigator). In addition to these inclusion criteria, either the survivor, the caregiver, or both the cancer survivor and caregiver must report at least mild anxiety (PROMIS distress-anxiety four-item raw score > 6) and/or mild depressive symptoms (PROMIS distress-depression four-item raw score > 6) to be enrolled into the study. For eligible consenting participants, interventions start approximately 2 weeks after the initial screening.

Participants for the study are being recruited from local cancer advocacy organizations located in metropolitan Tucson, Arizona, USA, as well as the University of Arizona Cancer Center (UACC) located in Tucson. Participants will receive compensation for their participation in the study: 20 USD for each assessment visit they complete, and 20 USD for each intervention session they attend.

### Study setting

All procedures for the proposed research (except saliva collection) are completed at the University of Arizona College of Nursing, including blood draws, self-report assessments, and the 8-week intervention classes (CBCT and CHE). Saliva (see biomarker methods below) is collected in participants’ homes.

### Study outcomes (measures)

**Fig. 2:**
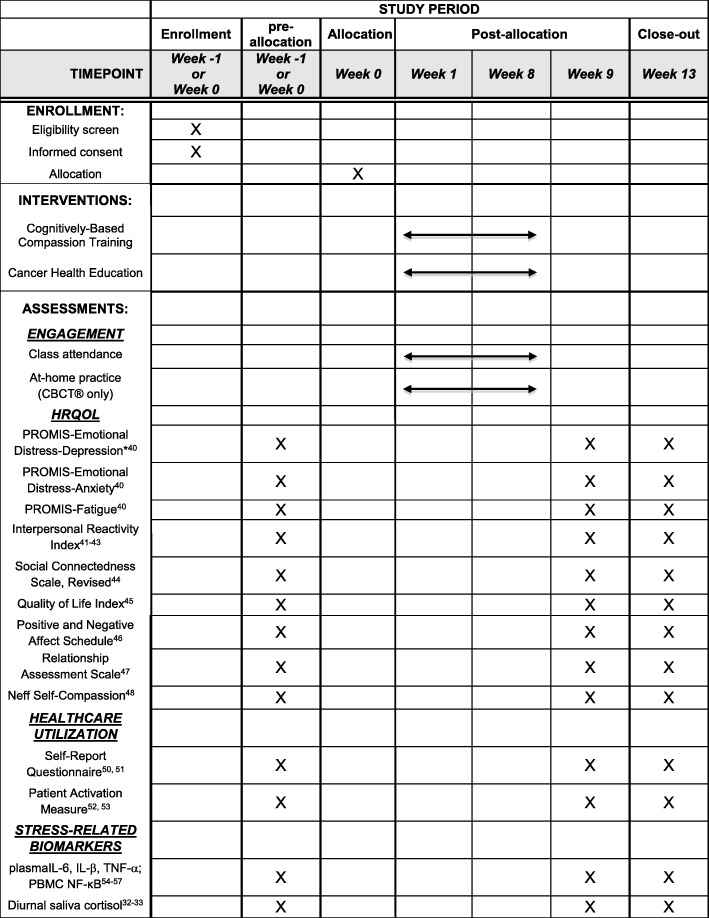
Study schedule of events. *Primary outcome. CBCT® Cognitively-Based Compassion Training, CHE cancer health education, IL interleukin, NF-κB nuclear factor-κB, PBMC peripheral blood mononuclear cell, TNF tumor necrosis factor

For a complete overview of study outcomes according to study visit schedule, please see Fig. 2 (arranged according to SPIRIT guidelines) [32, 33, 40–57].

#### Intervention engagement/acceptability

Intervention engagement and acceptability will be measured weekly at each CBCT and CHE session by adherence to intervention engagement requirements (class attendance, home practice (CBCT only)) by both dyad members. At-home practice will be recorded with a structured paper form, divided into days of the week, asking about practice time on a given day, and personal reflections of each practice. To support accurate documentation of at-home practice, and based on our prior experience with breast cancer survivors, we will use text, email, and telephone reminders to accurately complete the form shortly after completing a practice session [30]. We will also document rates of consent (before allocation) and retention of dyads (attending the majority of intervention classes and completion of all study assessment time points).

#### Health-related quality of life

Assessments of HRQOL defined in the study aims will be measured with several quantitative scales (see Table 1) [40–49]. Paper questionnaire forms will be inspected for completeness by study staff immediately after they are finished by participants, and participants will be asked to respond to any items that are left blank.

**Table 1:** Health-related quality of life assessments

	Instrument name	# items	Response options	Other information
Depression	PROMIS short-form Depression-8a^a^	8	Never, rarely, sometimes, often, always	Alpha 0.92; developed with IRT [40]
Anxiety	PROMIS short-form Anxiety-8	8	Never, rarely, sometimes, often, always	Very good reliability; developed with IRT [40]
Fatigue	PROMIS short-form 7-item Fatigue	7	Never, rarely, sometimes, often, always	Alpha 0.87; developed with IRT [40]
Empathy	Interpersonal Reactivity Index	28	5 responses from “does not describe me well” to “describes me very well”	Four subscales: perspective-taking, empathic concern, personal distress, and fantasy); good reliability [41–43]
Social connection	Social Connectedness Scale, Revised	20	6 responses from “strongly agree” to “strongly disagree”	Alpha 0.92 [44]
Global health-related quality of life	Quality of Life Index	60	6 responses from “very satisfied” to “very dissatisfied”, or from “very important” to “very unimportant”	Alpha 0.87–0.97 (cancer survivors), 0.92–0.93 (informal caregivers) [45]
Positive affect	Positive and Negative Affect Schedule	20	Very slightly or not at all, a little, moderately, quite a bit, and extremely	Alpha 0.87 (positive scale) [46]
Dyadic function	Relationship Assessment Scale	7	5 responses from “low satisfaction” to “high satisfaction”	Alpha 0.86 [47]
Self-compassion	Neff Self-Compassion Scale	26	5 responses from “almost never” to “almost always”	Alpha 0.92 [48, 49]

^a^Primary outcome

#### Healthcare utilization

Healthcare utilization will be measured using the following self-report assessments.

### Healthcare utilization self-report questionnaire

This assessment, designed by us, captures appointment keeping for oncologic, primary care, and noncancer specialty care (if needed), and closely follows approaches used in previous studies of people who have cancer and/or other chronic conditions [50, 51].

### Patient activation

We will assess patient activation using the short version of the Patient Activation Measure (PAM). The PAM consists of 13 items that form an interval level, unidimensional, Guttman-like scale to assess Hibbard’s four stages of activation [52, 53]. Items are statements about confidence, beliefs, knowledge, and skills about managing one’s health, with responses as degrees of agreement or disagreement. The PAM has been shown to have strong psychometric properties and is predictive of most health behaviors and many health outcomes [52, 53].

#### Stress-related biomarkers

##### Inflammatory biomarkers

Plasma and PBMCs will be obtained from blood drawn at all study assessment time points (T1, T2, and T3; see below) between the hours of 13:00 and 16:00 by venipuncture into EDTA-coated vacutainers. Plasma will be obtained by centrifugation, and PBMCs obtained by Ficoll-Paque gradient separation. Concentrations of IL-6, IL-β, and TNF-α in plasma will be batch analyzed using a high-sensitivity magnetic bead multiplex from R&D Systems. The PI’s laboratory has experience with multiplex assays for cytokines and has found good agreement between cytokine concentrations determined using multiplex assays and high-sensitivity enzyme-linked immunosorbent assays (ELISAs) [54]. NF-κB will be batch analyzed in PBMCs collected at each of the study time points using methods already established in the PI’s laboratory [55–57]. PBMCs will undergo nuclear extraction procedures with a high salt/low salt treatment before quantification of NF-κB binding to its DNA consensus sequence using a chemiluminescent transcription factor assay (Active Motif, Carlsbad, CA, USA). Of note, we are not analyzing gene expression or genetic polymorphisms in this project. The NF-κB assay assesses activation of this inflammatory signaling pathway by measuring the degree to which its activated form binds to a synthetic consensus sequence.

##### Diurnal saliva cortisol

Participants will be asked to collect their own saliva in the home setting at all study assessment time points. Concentrations of cortisol and C-reactive protein (CRP) in saliva will be determined using ELISA kits from Salimetrics (State College, PA, USA) according to the manufacturer’s instructions from saliva samples collected using the Sarstedt Salivette (Sarstedt, Nümbrect, Germany). The PI’s laboratory has experience using both of these analytic techniques [32, 33]. Participants will be provided with saliva collection kits that will include detailed instructions to collection saliva immediately on waking, and then in the evening about 2 h before bed time. Saliva samples collected for this study will not be analyzed for any purposes other than those stated immediately above.

### Interventions

#### Cognitively-Based Compassion Training

CBCT was designed at Emory University by one of the authors (LTN), and is a secular adaptation of techniques derived from traditional Tibetan Buddhist methods for cultivating compassion known as *lo-jong* [58]. The first term, *lo* or “mind”, refers to subjectivity, and *jong* refers to transformation or reorientation. The goal of this “transformation of subjectivity” is to temper egoistic self-centeredness (“self-cherishing”) toward altruism (“other-cherishing”) [59]. Throughout CBCT, topics and meditation practices build upon one another and are designed to develop an awareness of perceptions and attitudinal biases that can result in self-preoccupation and self-centeredness, challenge unexamined attitudes and cognitions toward other people, facilitate cognitive reappraisal to understand individuals and distress within a broader perspective of the human condition, and stimulate corrective affective experiences using visualizations and imagery during guided meditations. Putatively, the process is designed to reverse, or decondition, negatively valanced thoughts, emotions, and behaviors harmful to oneself and others, and to recondition (“transform”) them into those that are more prosocial and beneficial to oneself and others. Moving from self-centeredness to compassion does not occur rapidly, however. Therefore, CBCT follows a step-by-step process toward the final goal of inclusive and engaged compassion.

Over the course of 8 weeks there will be a total of eight CBCT sessions, one per week, led by the instructor (SED) who is certified by the CBCT training program at Emory University. Participating dyads will attend the weekly CBCT classes together, which we believe will be critical to make concepts salient for both the survivor and caregiver in each dyad. Each weekly session will last for about 120 min and will begin with a brief welcome meditation. This will be followed by a didactic phase in which the instructor will articulate goals and content of the current week, followed by a group discussion facilitated by the instructor. Group discussions will center on the challenges of cancer, cancer survival, and caregiver responses. All sessions combine lecture, discussion, experiential exercises, and reflective practice. Session will end with a 20–30 min meditation guided by the instructor and specific to the topic of the week. In between weekly meetings, dyads will be encouraged to practice a minimum of 10 min per day at home, and together as a dyad if possible, using the provided audio recording.

A study coordinator will call participants once per week, to remind them to practice at home, and also to attend the next CBCT class. Our prior work suggests that participants who practice an average of 3.1 sessions per week (10 min minimum per session) tend to exhibit meaningful changes in various outcomes including depressive symptoms, self-compassion, and inflammatory markers. Home practice will be encouraged by providing dyads with printed narrative summaries of weekly lessons, as well as guided meditation audio recordings developed by the Emory CBCT program, and applied for the current trial by the CBCT instructor (SED). Each week, participants will be asked to complete a CBCT practice log to assess their home practice, and to gather information on their engagement with CBCT. The course is divided into six modules, taught across eight sessions that meet for no more than 120 min, plus time for personal practice between sessions. All sessions combine lecture, discussion, experiential exercises, and reflective practice.

##### Week 1: resting in a moment of nurturance and developing attentional stability and clarity (module I)

Physical relaxation and focused attention training aid in mental stability and attention regulation and lead to concentration and later clarity of mental contents, states, and processes. Week 1 reviews confidentiality, defines compassion and its benefits, provides instruction in correct meditation posture, diaphragmatic breathing, and relaxation of muscle tension. This is followed by visualizing a remembered moment of nurturance that recalls the experience of compassion by a caregiver or caring other to prime the practitioner in feelings of safety and security. Last, the first mindfulness meditation practice (*shamatha*) is introduced and uses the breath as an object to focus attention for increasingly longer periods of time. These opening practices are the starting point of all subsequent CBCT meditation practice.

##### Week 2: insight into the nature of mental experience (module II)

The felt experience of compassion is one of kindness, connection, and unconditional positive regard. Cognitions, often with a negative emotional charge, are impermanent and transient, change rapidly, and can be observed as such. Week 2 reiterates the opening practices of the first week, and introduces the second mindfulness meditation practice (*vipassana*) to develop open awareness of subjective experience through nonjudgmental, nonreactive observation of the fluctuating (and impermanent) nature of thought, sensory experience, and emotion. This practice aids the participant in distinguishing internal mental experiences and external reality and creates a greater gap between an experience and the reaction to that experience, thus allowing for more deliberate choice in response or behavior. The technique is included in all subsequent CBCT meditation practice.

##### Week 3: self-compassion (module III)

Before compassion can be expressed to others, CBCT holds that one must first understand and reduce causes of distress in oneself, as well as attune to a sustained attitude of kindness toward the self. The assumption that all people share the common human desires for happiness, well-being, and freedom from distress is discussed, yet both external and internal conditions can interfere. Thus, causes of both external and internal interference need to be identified, along with learned perceptions and cognitive appraisals that accompany them. Concordant attitudes (e.g., narrow self-centeredness and self-preoccupation) and behaviors (e.g., (egoic) attachment and aversion, rumination, addiction, and avoidance) that can sustain faulty appraisals and that are potentially harmful require insight, reappraisal, and corrective emotional experience. Introspection, resolve, and commitment are then required for ongoing improvement. Week 3 introduces analytic meditation practice (as opposed to mindfulness practice) to identify and assess conditioned and habitual patterns of cognitions and appraisals contributing to distress to promote a resolution to correct these habits as they are identified.

##### Week 4: developing equanimity and impartiality (module IV)

CBCT holds that, in the universal desire for happiness, well-being, and freedom from distress, all individuals are alike; there are no differences among them (common humanity). Partiality and bias not only harm those regarded as adversaries or enemies, but also those regarded as loved ones since bias ultimately distorts interactions with others. Week 4 counters the participants’ learned attitudes of prejudice and partiality through continuing reflection on the common human desires for well-being and freedom from distress. Through guided analytical meditation practice, recognition of this commonality is promoted by visualizing people in the categories of friend, adversary, and stranger, with the goal of increasing identification with them (and thus empathic understanding) and of reducing indifference or excessive liking or disliking of some over others.

##### Week 5: appreciation and gratitude for others (module V)

CBCT holds that all people exist in an interconnected system, a web of interdependence for all needed resources and benefits. Recognition of this interdependence, in particular the high degree of dependence one’s well-being has on the efforts of others, decreases perceptions of interpersonal distance and social isolation (disconnection) and can lead to a sense of appreciation and gratitude for the beneficence of others, both familiar and unknown, as well as the hidden benefits that are often derived from adversaries. Week 5 explores interconnection and interdependence and the appreciation of others. Analysis and reflection during meditation examine the benefits received from others, even those received from adversaries. Further meditation practice guides the participant in visualizing extending appreciation and gratitude to an ever-widening circle of others.

##### Week 6: affection (module V)

The CBCT model contends that experiences of appreciation and gratitude lead to feelings of endearment, or warm affection. Warmth, together with identification (see module IV), stimulates empathy, or the recognition that others, too, experience distress and its causes. Week 6 introduces affection for others based on their beneficence and also their similarity to oneself in wanting well-being yet often experiencing distress. Relating to others with profound affection and endearment then becomes the preconditions for empathy; in turn, empathy can catalyze compassion. Meditative reflection is guided to recall the kindnesses of others, their similarities to oneself (“just like me”), and to strengthen endearment and affection towards them. Further meditative reflection is guided on the drawbacks of egoistic attitudes to lessen self-centeredness.

##### Week 7: empathetic concern and engaged compassion (module VI)

Once insight into the causes of distress (week 3) is combined with affection toward and empathic understanding of others (week 6), CBCT holds that compassion naturally ensues. Week 7 focuses on sustaining the spontaneous empathetic concern that naturally arises when these two conditions are present: 1) holding someone with deep affection and warm-heartedness; and 2) attuning to their dissatisfaction, distress, and vulnerability. To sustain the emerging motivation to alleviate their distress (while simultaneously acknowledging one’s limitations and boundaries via self-compassion), week 7 provides instruction to attune to this compassionate love as an image of energy or light, radiating outward and including first our family and friends and then increasingly others. Instruction guides the practitioner in wishing compassion as a desirable thought of “how *wonderful* it would be if others were happy and free from dissatisfaction”.

##### Week 8: empathic concern and engaged compassion (module VI)

When a genuine desire for compassion is deepened and accompanied with a determined motivation to help others when possible, the final step of CBCT is considered to be activated or engaged compassion. Continued practice helps strengthen all of the prior skills and insights meant to support an embodied compassionate responsiveness toward others. In week 8, class exercises promote group closure, and a final comprehensive meditation practice is conducted. The same guided meditation is used as in week 7, but more emphasis during reflection is placed on phrases that promote a move from simply wishing others happiness and freedom from distress to a motivational readiness to assist whenever possible (“*may* they be happy and free from dissatisfaction”).

#### Cancer health education

The cancer health education (CHE) intervention is an adaptation of the in-person attention control called Health Discussion, a protocol used previously by our group (LTN and TWWP) [34] that incorporates select components from a telephone-based health education program also used previously by us (TAB and CS) [60, 61]. CHE focuses on topics relevant to health and cancer for survivors and caregivers including cancer advocacy, health and cancer biology, nutrition, lifestyle interventions such as physical activity and goals for physical activity, the importance of good sleep, the impact of stress, and mental health and social support.

Over the course of 8 weeks there will be a total of eight sessions, one session per week, similar to CBCT. As with CBCT, each session will last for approximately 120 min and will include a combination of lecture, group discussion, and experiential exercises. CHE is a good control condition for a trial investigating the benefits of CBCT for several reasons. First, like CBCT, CHE involves building new social contacts while also discussing and learning new topics relevant for solid tumor cancer survivors and their caregivers. Second, also like CBCT, CHE requires individuals to travel to a central location to receive the intervention. However, unlike CBCT, CHE does not involve training around meditation, or structured discussions about compassion or kindness.

##### Week 1: cancer advocacy

This module begins with a discussion on cancer advocacy that includes the definition of cancer advocacy, how to be an advocate for your own cancer care throughout survivorship, how to be an advocate for others with cancer, and how to approach public interest advocacy for cancer. This module also discusses current events related to cancer, trends about cancer diagnoses, and the latest science and research about cancer (e.g., the Cancer Moonshot). At the conclusion of this module participants will have a broad understanding of the major themes of cancer advocacy and the significance of cancer advocacy for promoting the wellness of the self, family, and society, and cancer research.

##### Week 2: health through the lifespan

This module provides an overview of the biology of cancer, how cancer is defined, how cancer is treated, and the side effects of cancer treatments. Also reviewed are topics relevant to maintaining a healthy lifestyle over the lifespan including leading causes of mortality besides cancer, brain and mental health, and health screenings. At the conclusion of this module participants will be familiar with the leading causes of death and avoidable causes of death, the role of general health habits, the importance of health screenings, and the relevance of mind-body connections for health and cancer survivorship.

##### Weeks 3 and 4: nutrition

The first week of this module focuses on basic components of food such as carbohydrates, proteins, and fat. Whole grains, hydration, and caffeine are also discussed, as well as essential nutrients and dietary fiber. The second week provides an overview of healthy diet tips, serving sizes, and factors that contribute to unhealthy eating. Key topics in the second week include nutrition related to obesity, healthy serving sizes, nutritional “trade-offs”, healthy grocery shopping, and tips for maintaining a healthy diet. At the conclusion of this module participants will have an understanding of food components, as well as basic strategies to maintain good nutrition to promote health, including in cancer survivorship.

##### Week 5: physical activity

This module reviews the importance of physical activity in survivorship and also the importance of physical activity for the wellness of noncancer survivors across the lifespan. This module provides an overview and explanation of the basic components, principles, and health benefits of physical fitness. Information for developing and implementing a personal fitness plan and goals are also presented. The module concludes with practical tips for exercise, for example staying fit while traveling. It will also address the consequences of a lack of exercise (e.g., obesity), the health benefits of exercise (e.g., mental wellness, healthy aging, cardiovascular wellness), lactic acid, muscle burning and soreness, planning an exercise schedule, and exercise nutrition. At the conclusion of this module participants will have an understanding of exercise basics, the relationship between exercise and wellness, and how to better incorporate exercise in their own lives.

##### Week 6: sleep

This module provides an overview of the sleep cycle, the benefits of sleep, common sleep disorders, and tips for better sleep. Topics considered also include the neuroscience mechanisms of sleep, common sleep disorders, and tips for diet to promote healthy sleep, including for survivors. By the end of the module participants will understand the mechanisms of sleep, why sleep is important for good health (including in survivorship), and how to get better sleep.

##### Week 7: stress

This module reviews the concept of stress and how stress is known to impact health, including the biological mechanisms involved. Topics discussed include the definition of stress, a review of “America’s most stressed out cities”, types of stress (distress and eustress), negative effects of chronic stress, and basic ways to cope with stress. Of note, this module will not provide participants with a comprehensive stress management plan. Instead, the coping strategies for stress considered will be limited to general topics such as good sleep, physical activity, and diet (to synergize with earlier CHE modules).

##### Week 8: mental health and social support

This module provides a broad overview of mental health disorders (e.g., depression, anxiety disorders, eating disorders), as well as disorders that many survivors and caregivers experience. The health-related consequences of loneliness and the benefits of social interaction are also reviewed. This module also introduces the concept of social capital and explores the impact of social networking sites. By the end of the module participants will have a general understanding of mental health disorders and how social contact can promote wellness for survivors and caregivers.

### Power, data management plan, and analytic plan

As a preliminary effort, this study’s sample size was not determined based on power considerations. The goal is to estimate the effect sizes for the differences between the CBCT and CHE in the specified outcomes at weeks 9 and 13, so that the next definitive trial can be formally powered using the effect sizes estimated in this pilot study. Therefore, the sample size for this study (*n* = 40 dyads (80 participants total); CBCT *n* = 20, CHE attention control *n* = 20) is based on the number of cancer survivor-caregiver dyads projected to meet inclusion criteria and complete the entire study protocol in the study timeframe. The estimate of the effect size will be used to power the definitive trial, provided that the estimate falls into the range of clinically significant differences of 1/3 of the standard deviation or greater [62, 63]. The rigor of the proposed pilot randomized controlled trial design (i.e., having the CHE attention control) will secure the attribution of the effects to the intervention.

Questionnaire total scores, biomarker data, and other relevant endpoints (e.g., class attendance) will be entered into the study database and managed in SPSS (IBM Analytics, Armonk, NY, USA). Intervention fidelity will be summarized using the number of completed sessions.

For Aim 1 analysis (Hypothesis 1), acceptability will be measured by attendance at weekly classes. Acceptability will be determined as > 75% average attendance at weekly CBCT classes and > 75% average attendance at weekly CHE classes. For the other aims, both unadjusted and adjusted effect sizes will be estimated. Estimates of the unadjusted effect sizes will be computed as differences between sample means of two study groups at each time point divided by the pooled standard deviation. Furthermore, to reflect longitudinal design and inform planning of the larger definitive trial, we will estimate the effect sizes adjusted for repeated measures and inclusion of covariates. For Aim 2 analysis, two repeated measures of HRQOL outcomes will be entered into a linear mixed effects model with the following covariates: outcome measure at baseline, study group, time (week 9 or week 13), and group by time interaction. The least square (LS) means according to the levels of the interaction term will be output from the model, and effect sizes at each time point will be estimated as differences between the LS means divided by the adjusted standard deviation. Separate models will be fit for each survivor outcome and each caregiver outcome. We will use the same approach to estimate effect sizes for stress-related biomarker outcomes and associated hypotheses (Aim 3). A similar approach will also be employed in the analysis for Aim 4 to estimate effect sizes for healthcare utilization-related outcomes, except that generalized linear mixed effects modeling will be used for the analysis of two repeated measures, with the appropriate error distributions specified based on observed distributions of different health service use outcomes (counts of health services use, kept appointments, and patient activation).

### Data handling

All study material including source documents and casebooks will be stored in a secured room at the University of Arizona College of Nursing or on a secure computer server or personal computer that are compliant with the United States Health Insurance Portability and Accountability Act (HIPAA) and that can only be accessed by study researchers. Data collected and study forms completed during assessment visits will be stored by study number, not by subject name. A paper copy list linking subject names to their subject numbers will be stored separately from all other study documents. Study materials including electronic data and study consent forms will be maintained for 6 years after the conclusion of the project. Aggregated results will be presented at professional conferences, published in appropriate scholarly journals, and provided to study participants upon request. The study Principal Investigator as well as co-Investigators will have access to the final study dataset, and no contracts are required to regulate this access.

### Data monitoring

It is possible that some of the participants may develop psychiatric and/or medical problems from screening to enrollment to after study end (up to 3 months later). Also, although not expected, it is possible that blood draws may result in adverse events for some participants. We have therefore elected to use the Data Safety Monitoring Board (DSMB) at the University of Arizona College of Nursing for this study. This DSMB will provide independent oversight to further promote the protection of human subjects and the overall integrity of the study. Once every 6 months, the DSMB will review a report from the study that includes: 1) the number of participants who signed consent for the study; 2) the number of dropouts; 3) reasons for these dropouts; 4) any safety concerns or adverse events (i.e., solicited and spontaneously reported adverse events and other unintended effects of trial interventions or trial conduct); 5) an up-to-date consent form; and 6) measures taken to protect confidentiality (e.g., data storage, use of coded ID numbers, etc.). After reviewing this information, the DSMB will issue its own report summarizing any serious and unexpected adverse events or other unanticipated problems that involve risk to study participants, and whether these appear related to the study-based research assessment protocol. Given the nature of the intervention and active attention control group, this study does not have a plan for interim analyses. Analyses guided by hypotheses noted above will only take place after all participants have completed the study protocol.

## Discussion

This trial will be the first to determine acceptability of a compassion meditation-based intervention (CBCT) in dyads consisting of solid tumor cancer survivors and their caregivers. We believe the trial will be the first step toward demonstrating that CBCT is especially worthwhile for cancer survivor-caregiver dyads for several reasons. First, CBCT has already been shown to improve various dimensions of HRQOL for breast cancer survivors [30]. Second, both impairments in HRQOL and affect have been found to be interdependent between cancer survivors and their caregivers [25, 26, 64] and, if CBCT improves HRQOL in one dyadic member, it may transfer to the other member. Third, unlike other meditation programs that only include mindfulness, CBCT actively incorporates the intellectual analysis of how individuals are interconnected with others while encouraging a move away from egoistic self-centeredness and preoccupation. Thus, among meditation programs available for cancer survivors and their caregivers, CBCT is likely very well suited to encourage healthy interconnectedness between cancer survivors and their caregivers to improve HRQOL of the dyad. The current trial is also poised to be a first step toward revealing an interdependence in stress-related biomarkers within the dyad (with a fully powered trial), including their change over the course of the interventions. To the best of our knowledge, an interdependence of cortisol or inflammatory biomarkers has never been demonstrated before in cancer survivor-caregiver dyads.

Considerable evidence supports the inclusion of stress-related biomarkers in the current pilot study. Breast cancer survivors have been found to have increased circulating concentrations of key inflammatory biomarkers that positively correlate with fatigue [65–71]. Severity of major depression features have also been associated with inflammatory biomarkers, including IL-6, in cancer survivors [72], and our group has found that NF-κB activation in circulating immune cells is elevated in breast cancer survivors and is positively associated with fatigue and depression [55]. With respect to HRQOL impairments of caregivers, there is also a rich science on how inflammation is causally linked with quality of life impairments experienced by noncancer survivors [73, 74], although research that is focused exclusively on caregivers of cancer survivors is not conclusive [22, 75, 76]. For cortisol, significant distress in breast cancer survivors has been associated with dysregulation of the hypothalamic-pituitary adrenal axis, most frequently manifested as reduced variation in cortisol rhythm from morning (when cortisol is normally high) to evening (when cortisol is normally low) [77]. Psychological distress has been found to be associated with flattening of the diurnal cortisol slope in numerous patient populations, including those with cancer [78–81]. Previous research has documented that a flatter cortisol slope, or abnormal diurnal cortisol rhythm, has been associated with fatigue [82], tumor progression and reduced survival [83, 84], depression [80, 85], and negative psychosocial outcomes in breast cancer survivors [86–89].

There is also a strong rationale to assess healthcare utilization in the current study, especially the dimension of patient activation. Activated patients are those with the motivation, knowledge, skills, and confidence to make effective decisions in managing their health. Those with low activation are typically passive recipients of care and do not believe in the need for an active patient role. Those who are highly activated are proactive about their health and engage in many recommended health behaviors. Activation, however, is not either/or. A widely used developmental model of activation involves four stages: 1) believing the patient role is important; 2) having the confidence and knowledge necessary to take action; 3) actually taking action to maintain and improve one’s health; and 4) staying the course even under stress [52, 53]. Correlational studies have found patient activation to be related to healthy lifestyle behaviors (e.g., physical activity, eating fruits and vegetables), appropriate use of healthcare services (e.g., having fewer hospitalizations), and self-management of chronic conditions (e.g., diabetic eye examinations, recording blood pressure readings, HbA1c control). Other studies show patient activation to be a modifiable characteristic, and that interventions can increase self-management capacities and health service use.

There are several practical and operational challenges worth noting here that have already been encountered by this research team. First, unlike other wellness programs for cancer survivors, CBCT critically involves the coparticipation of a caregiver. Although for many cancer survivors a caregiver may be easy to name, and easy to “bring along” for many cancer survivors, for other survivors there are scheduling problems or the caregiver changes his/her mind about participating which can affect the effectiveness of CBCT for the dyad. We are actively working to make the trial available to as many dyads as possible by scheduling sessions at times that work best for all, and if a survivor is unable to name a caregiver, we do not enroll them but refer them to other wellness resources. Second, based on our experience with research involving CBCT in other populations (e.g., healthy community-dwelling adults, foster care children), maintaining engagement of dyads randomized to CHE may be a challenge. We have therefore included a complimentary CBCT course for all dyads randomized to CHE shortly after the last study assessment time point to encourage study participation.

In summary, the trial presented here will elucidate whether CBCT is acceptable and feasible for cancer survivor-caregiver dyads, and if CBCT is associated with improvements in HRQOL measures, biomarkers of inflammation, cortisol diurnal rhythm, and healthcare utilization. The work in this trial will result in a larger, well-powered trial and may result in the addition of CBCT to supportive oncology care programs to improve HRQOL in both members of the survivor-caregiver dyad.

## Trial status

The protocol date is 30 March 2018 and is currently approved by the Human Subjects Protection Program at the University of Arizona (1618 E. Helen St., P.O. Box 245,137, Tucson, AZ 85724–5137, USA; tel. 520 626 6721). The original protocol approval date was 1 December 2017. Recruitment for the trial began on 14 March 2018, after first posting on clinicaltrials.gov as NCT03459781. At the time this report was submitted the trial was recruiting participants. Recruitment as well as all other procedures for the trial is anticipated to conclude by September 2019. This study protocol reports study protocol version 1.2, which includes stress-related biomarkers.

## Additional file

Additional file 1: SPIRIT 2013 checklist: recommended items to address in a clinical trial protocol and related documents. (DOC 121 kb)

## Abbreviations

CBCT: Cognitively-Based Compassion Training
CHE: Cancer health education
HRQOL: Health-related quality of life
IL: Interleukin
NF-κB: Nuclear factor-κB
PBMC: Peripheral blood mononuclear cell
TNF: Tumor necrosis factor

## References

[1] NCI. SEER cancer statistics review (CSR) 1975–2014. 2017; https://seer.cancer.gov/csr/1975_2014/. Accessed 1 May 2018, 2015.

[2] Tung HY, Chao TB, Lin YH, Wu SF, Lee HY, Ching CY, Hung KW, Lin TJ (2016). Depression, fatigue, and QoL in colorectal cancer patients during and after treatment. West J Nurs Res.

[3] Watts S, Leydon G, Birch B, Prescott P, Lai L, Eardley S, Lewith G (2014). Depression and anxiety in prostate cancer: a systematic review and meta-analysis of prevalence rates. BMJ Open.

[4] Fann JR, Thomas-Rich AM, Katon WJ, Cowley D, Pepping M, McGregor BA, Gralow J (2008). Major depression after breast cancer: a review of epidemiology and treatment. Gen Hosp Psychiatry.

[5] Krebber AM, Buffart LM, Kleijn G, Riepma IC, de Bree R, Leemans CR, Becker A, Brug J, van Straten A, Cuijpers P, Verdonck-de Leeuw IM (2014). Prevalence of depression in cancer patients: a meta-analysis of diagnostic interviews and self-report instruments. Psychooncology.

[6] Schneider EC, Malin JL, Kahn KL, Ko CY, Adams J, Epstein AM (2007). Surviving colorectal cancer: patient-reported symptoms 4 years after diagnosis. Cancer.

[7] Colloca G, Venturino A, Governato I, Checcaglini F (2016). Incidence and correlates of fatigue in metastatic castration-resistant prostate cancer: a systematic review. Clin Genitourin Cancer.

[8] Hofman M, Ryan JL, Figueroa-Moseley CD, Jean-Pierre P, Morrow GR (2007). Cancer-related fatigue: the scale of the problem. Oncologist.

[9] Lawrence DP, Kupelnick B, Miller K, Devine D, Lau J (2004). Evidence report on the occurrence, assessment, and treatment of fatigue in cancer patients. J Natl Cancer Inst Monogr.

[10] Curt GA, Breitbart W, Cella D, Groopman JE, Horning SJ, Itri LM, Johnson DH, Miaskowski C, Scherr SL, Portenoy RK, Vogelzang NJ (2000). Impact of cancer-related fatigue on the lives of patients: new findings from the Fatigue Coalition. Oncologist.

[11] Saboonchi F, Petersson LM, Wennman-Larsen A, Alexanderson K, Vaez M (2015). Trajectories of anxiety among women with breast cancer: a proxy for adjustment from acute to transitional survivorship. J Psychosoc Oncol.

[12] Maass SW, Roorda C, Berendsen AJ, Verhaak PF, de Bock G (2015). The prevalence of long-term symptoms of depression and anxiety after breast cancer treatment: a systematic review. Maturitas.

[13] Eisenberg SA, Kurita K, Taylor-Ford M, Agus DB, Gross ME, Meyerowitz BE (2015). Intolerance of uncertainty, cognitive complaints, and cancer-related distress in prostate cancer survivors. Psychooncology.

[14] Lowery AE, Krebs P, Coups EJ, Feinstein MB, Burkhalter JE, Park BJ, Ostroff JS (2014). Impact of symptom burden in post-surgical non-small cell lung cancer survivors. Support Care Cancer.

[15] Custers JA, Gielissen MF, Janssen SH, de Wilt JH, Prins JB (2016). Fear of cancer recurrence in colorectal cancer survivors. Support Care Cancer.

[16] Braamse AM, van Turenhout ST, Terhaar Sive Droste JS, de Groot GH, van der Hulst RW, Klemt-Kropp M, Kuiken SD, Loffeld RJ, Uiterwaal MT, Mulder CJ, Dekker J (2016). Factors associated with anxiety and depressive symptoms in colorectal cancer survivors. Eur J Gastroenterol Hepatol.

[17] Lambert SD, Girgis A, Lecathelinais C, Stacey F (2013). Walking a mile in their shoes: anxiety and depression among partners and caregivers of cancer survivors at 6 and 12 months post-diagnosis. Support Care Cancer.

[18] Cubukcu M (2018). Evaluation of quality of life in caregivers who are providing home care to cancer patients. Support Care Cancer.

[19] Kent EE, Rowland JH, Northouse L, Litzelman K, Chou WY, Shelburne N, Timura C, O’Mara A, Huss K (2016). Caring for caregivers and patients: research and clinical priorities for informal cancer caregiving. Cancer.

[20] Northouse L, McCorkle R, Holland JCBW, Butow PN, Jacobsen PB, Loscalzo MJ, McCorkle R (2015). Spouse caregivers of cancer patients. Psycho-oncology.

[21] Litzelman Kristin, Kent Erin E., Mollica Michelle, Rowland Julia H. (2016). How Does Caregiver Well-Being Relate to Perceived Quality of Care in Patients With Cancer? Exploring Associations and Pathways. Journal of Clinical Oncology.

[22] Sherwood PR, Price TJ, Weimer J, Ren D, Donovan HS, Given CW, Given BA, Schulz R, Prince J, Bender C, Boele FW, Marsland AL (2016). Neuro-oncology family caregivers are at risk for systemic inflammation. J Neuro-Oncol.

[23] Kiecolt-Glaser JK, Preacher KJ, MacCallum RC, Atkinson C, Malarkey WB, Glaser R (2003). Chronic stress and age-related increases in the proinflammatory cytokine IL-6. Proc Natl Acad Sci U S A.

[24] Schulz R, Sherwood PR (2008). Physical and mental health effects of family caregiving. Am J Nurs.

[25] Segrin C, Badger TA (2014). Psychological and physical distress are interdependent in breast cancer survivors and their partners. Psychol Health Med.

[26] Segrin C, Badger TA, Harrington J (2012). Interdependent psychological quality of life in dyads adjusting to prostate cancer. Health Psychol.

[27] Hatfield E, Cacioppo JT, Rapson RL (1994). Emotional contagion.

[28] Ozawa-de Silva B, Negi LT, Singer T, Bolz M (2013). Cognitively-Based Compassion Training (CBCT): protocol and key concepts. Compassion: bridging practice and science.

[29] Ozawa-de Silva B, Dodson-Lavelle B (2011). Education of heart and mind: practical and theoretical issues in teaching Cognitively-Based Compassion Training to children. Practial Matters.

[30] Dodds SE, Pace TWW, Bell ML, Fiero M, Negi LT, Raison CL, Weihs KL (2015). Feasibility of Cognitively-Based Compassion Training (CBCT) for breast cancer survivors: a randomized, wait list controlled pilot study. Support Care Cancer.

[31] Mascaro JS, Rilling JK, Tenzin Negi L, Raison CL (2013). Compassion meditation enhances empathic accuracy and related neural activity. Soc Cogn Affect Neurosci.

[32] Pace TWW, Negi LT, Dodson-Lavelle B, Ozawa-de Silva B, Reddy SD, Cole SP, Danese A, Craighead LW, Raison CL (2013). Engagement with Cognitively-Based Compassion Training is associated with reduced salivary C-reactive protein from before to after training in foster care program adolescents. Psychoneuroendocrinology.

[33] Reddy SD, Negi LT, Dodson-Lavelle B, Ozawa-de Silva B, Pace TWW, Cole SP, Raison CL, Craighead LW (2012). Cognitively-based Compassion Training: a promising prevention strategy for at-risk adolescents. J Child Fam Stud.

[34] Desbordes G, Negi LT, Pace TWW, Wallace BA, Raison CL, Schwartz EL (2012). Effects of mindful-attention and compassion meditation training on amygdala response to emotional stimuli in an ordinary, non-meditative state. Front Hum Neurosci.

[35] Pace TWW, Negi LT, Sivilli TI, Issa MJ, Cole SP, Adame DD, Raison CL (2010). Innate immune, neuroendocrine and behavioral responses to psychosocial stress do not predict subsequent compassion meditation practice time. Psychoneuroendocrinology.

[36] Pace TWW, Negi LT, Adame DD, Cole SP, Sivilli TI, Brown TD, Issa MJ, Raison CL (2009). Effect of compassion meditation on neuroendocrine, innate immune and behavioral responses to psychosocial stress. Psychoneuroendocrinology.

[37] Dantzer R, O’Connor JC, Freund GG, Johnson RW, Kelley KW (2008). From inflammation to sickness and depression: when the immune system subjugates the brain. Nat Rev Neurosci.

[38] Bower JE (2014). Cancer-related fatigue—mechanisms, risk factors, and treatments. Nat Rev Clin Oncol.

[39] Cheruvu VK, Oancea SC (2016). Current depression as a potential barrier to health care utilization in adult cancer survivors. Cancer Epidemiol.

[40] Cella D, Riley W, Stone A, Rothrock N, Reeve B, Yount S, Amtmann D, Bode R, Buysse D, Choi S, Cook K, Devellis R, DeWalt D, Fries JF, Gershon R, Hahn EA, Lai JS, Pilkonis P, Revicki D, Rose M, Weinfurt K, Hays R (2010). The Patient-Reported Outcomes Measurement Information System (PROMIS) developed and tested its first wave of adult self-reported health outcome item banks: 2005-2008. J Clin Epidemiol.

[41] Pulos S, Elison J, Lennon R (2004). The hierarchical structure of the Interpersonal Reactivity Index. Soc Behav Pers.

[42] Davis MH (1983). Measuring individual differences in empahty: evidence for a multi-dimensional approach. J Pers Soc Psychol.

[43] Davis MH (1994). Empathy: a social psychological approach.

[44] Lee RM, Draper M, Lee S (2001). Social connectedness, dysfunctional interpersonal behaviors, and psychological distress: testing a mediator model. J Couns Psychol.

[45] Ferrans CE, Powers M. Ferrans and Powers Quality of Life Index (QLI): reliability and validity. [website]. https://qli.org.uic.edu/reliability/reliabilityhome.htm. Accessed 1 June 2018.

[46] Watson D, Clark LA, Tellegen A (1988). Development and validation of brief measures of positive and negative affect: the PANAS scales. J Pers Soc Psychol.

[47] Hendrick SS (1988). A generic measure of relationship satisfaction. J Marriage Fam.

[48] Neff KD (2003). Development and validation of a scale to measure self-compassion. Self Identity.

[49] Breines JG, Thoma MV, Gianferante D, Hanlin L, Chen X, Rohleder N (2014). Self-compassion as a predictor of interleukin-6 response to acute psychosocial stress. Brain Behav Immun.

[50] Lorig KR, Sobel DS, Ritter PL, Laurent D, Hobbs M (2001). Effect of a self-management program on patients with chronic disease. Eff Clin Pract.

[51] Kirchhoff AC, Lyles CR, Fluchel M, Wright J, Leisenring W (2012). Limitations in health care access and utilization among long-term survivors of adolescent and young adult cancer. Cancer.

[52] Hibbard JH, Stockard J, Mahoney ER, Tusler M (2004). Development of the Patient Activation Measure (PAM): conceptualizing and measuring activation in patients and consumers. Health Serv Res.

[53] Hibbard JH, Mahoney ER, Stockard J, Tusler M (2005). Development and testing of a short form of the patient activation measure. Health Serv Res.

[54] Quinn Andrea M., Williams Allison R., Sivilli Teresa I., Raison Charles L., Pace Thaddeus W. W. (2018). The plasma interleukin-6 response to acute psychosocial stress in humans is detected by a magnetic multiplex assay: comparison to high-sensitivity ELISA. Stress.

[55] Torres MA, Pace TWW, Liu T, Felger JC, Mister D, Doho GH, Kohn JN, Barsevick AM, Long Q, Miller AH (2014). Predictors of depression in breast cancer patients treated with radiation: role of prior chemotherapy and nuclear factor kappa B. Cancer.

[56] Pace TWW, Wingenfeld K, Schmidt I, Meinlschmidt G, Hellhammer DH, Heim CM (2012). Increased peripheral NF-kappaB pathway activity in women with childhood abuse-related posttraumatic stress disorder. Brain Behav Immun.

[57] Pace TWW, Mletzko TC, Alagbe O, Musselman DL, Nemeroff CB, Miller AH, Heim CM (2006). Increased stress-induced inflammatory responses in male patients with major depression and increased early life stress. Am J Psychiatry.

[58] Negi LT (2013). Emory compassion meditation protocol: Cognitively-Based Compassion Training manual.

[59] Ozawa-de Silva B, Dodson-Lavelle B, Raison CL, Negi LT (2012). Compassion and ethics: scientific approaches to the cultivation of compassion as a foundation for ethical subjectivity and well-being. J Healthcare Sci Human.

[60] Badger TA, Segrin C, Figueredo AJ, Harrington J, Sheppard K, Passalacqua S, Pasvogel A, Bishop M (2011). Psychosocial interventions to improve quality of life in prostate cancer survivors and their intimate or family partners. Qual Life Res.

[61] Badger TA, Segrin C, Hepworth JT, Pasvogel A, Weihs K, Lopez AM (2013). Telephone-delivered health education and interpersonal counseling improve quality of life for Latinas with breast cancer and their supportive partners. Psychooncology.

[62] Cella D, Eton DT, Fairclough DL, Bonomi P, Heyes AE, Silberman C, Wolf MK, Johnson DH (2002). What is a clinically meaningful change on the Functional Assessment of Cancer Therapy-Lung (FACT-L) questionnaire? Results from Eastern Cooperative Oncology Group (ECOG) study 5592. J Clin Epidemiol.

[63] Sloan JA, Cella D, Hays RD (2005). Clinical significance of patient-reported questionnaire data: another step toward consensus. J Clin Epidemiol.

[64] Segrin C, Badger TA, Sikorskii A, Crane TE, Pace TWW (2018). A dyadic analysis of stress processes in Latinas with breast cancer and their family caregivers. Psychooncology.

[65] Miller AH, Ancoli-Israel S, Bower JE, Capuron L, Irwin MR (2008). Neuroendocrine-immune mechanisms of behavioral comorbidities in patients with cancer. J Clin Oncol.

[66] Collado-Hidalgo A, Bower JE, Ganz PA, Irwin MR, Cole SW (2008). Cytokine gene polymorphisms and fatigue in breast cancer survivors: early findings. Brain Behav Immun.

[67] Bower JE (2005). Prevalence and causes of fatigue after cancer treatment: the next generation of research. J Clin Oncol.

[68] Bower JE, Ganz PA, Aziz N, Fahey JL (2002). Fatigue and proinflammatory cytokine activity in breast cancer survivors. Psychosom Med.

[69] Bower JE, Ganz PA, Tao ML, Hu W, Belin TR, Sepah S, Cole S, Aziz N (2009). Inflammatory biomarkers and fatigue during radiation therapy for breast and prostate cancer. Clin Cancer Res.

[70] Wratten C, Kilmurray J, Nash S, Seldon M, Hamilton CS, O’Brien PC, Denham JW (2004). Fatigue during breast radiotherapy and its relationship to biological factors. Int J Radiat Oncol Biol Phys.

[71] Collado-Hidalgo A, Bower JE, Ganz PA, Cole SW, Irwin MR (2006). Inflammatory biomarkers for persistent fatigue in breast cancer survivors. Clin Cancer Res.

[72] Musselman DL (2001). Higher than normal plasma interleukin-6 concentrations in cancer patients with depression: preliminary findings. Am J Psychiatr.

[73] Marsland AL, Walsh C, Lockwood K, John-Henderson NA (2017). The effects of acute psychological stress on circulating and stimulated inflammatory markers: a systematic review and meta-analysis. Brain Behav Immun.

[74] Howren MB, Lamkin DM, Suls J (2009). Associations of depression with C-reactive protein, IL-1, and IL-6: a meta-analysis. Psychosom Med.

[75] Miller GE, Murphy ML, Cashman R, Ma R, Ma J, Arevalo JM, Kobor MS, Cole SW (2014). Greater inflammatory activity and blunted glucocorticoid signaling in monocytes of chronically stressed caregivers. Brain Behav Immun.

[76] Bower JE, Ganz PA, Irwin MR, Arevalo JM, Cole SW (2011). Fatigue and gene expression in human leukocytes: increased NF-kappaB and decreased glucocorticoid signaling in breast cancer survivors with persistent fatigue. Brain Behav Immun.

[77] Spiegel D, Giese-Davis J (2003). Depression and cancer: mechanisms and disease progression. Soc Biol Psychiatry.

[78] Spiegel D, Giese-Davis J, Taylor CB, Kraemer H (2006). Stress sensitivity in metastatic breast cancer: analysis of hypothalamic-pituitary-adrenal axis function. Psychoneuroendocrinology.

[79] Giese-Davis J, Spiegel D. Emotional expression and cancer progression. In: Davidson RJ, Scherer K, Hill Goldsmith H, eds. Series in affective science. Handbook of affective sciences. New York, NY: Oxford University Press; 2003:1053–1082.

[80] Giese-Davis J, Wilhelm FH, Conrad A, Abercrombie HC, Sephton S, Yutsis M, Neri E, Taylor CB, Kraemer HC, Spiegel D (2006). Depression and stress reactivity in metastatic breast cancer. Psychosom Med.

[81] Giese-Davis J, Collie (2010). Decrease in depression symptoms is associated with longer survival in patients with metastatic breast cancer: a secondary analysis. J Clin Oncol.

[82] Bower JE, Ganz PA, Dickerson SS, Peterson L, Azia N, Fahey JL (2005). Diurnal cortisol rhythm and fatigue in breast cancer survivors. PsychoNeuroendocrinology.

[83] Sephton SE, Sapolsky RM, Kraemer HC, Spiegel D (2000). Diurnal cortisol rhythm as a predictor of breast cancer survival. J Natl Cancer Inst.

[84] Chida Y, Hamer M, Wardle J, Steptoe A (2008). Do stress-related psychosocial factors contribute to cancer incidence and survival?. Nat Clin Pract Oncol.

[85] Jarcho MR, Slavich GM, Tylova-Stein H, Wolkowitz OM, Burke HM (2013). Dysregulated diurnal cortisol pattern is associated with glucocorticoid resistance in women with major depressive disorder. Biol Psychol.

[86] Spiegel D, Giese-Davis J, Taylor CB, Kraemer H (2006). Stress sensitivity in metastatic breast cancer: analysis of the hypothalmic-pituitary-adrenal axis function. PsychoNeuroendocrinology.

[87] Giese-Davis J, Sephton SE, Abercrombie HC, Duran RE, Spiegel D (2004). Repression and high anxiety are associated with aberrant diurnal cortisol rhythms in women with metastatic breast cancer. Health Psychol.

[88] Abercrombie HC, Giese-Davis J, Sephton S, Epel ES, Turner-Cobb JM, Spiegel D (2004). Flattened cortisol rhythms in metastatic breast cancer patients. Psychoneuroendocrinology.

[89] Turner-Cobb JM, Sephton SE, Koopman C, Blake-Mortimer J, Spiegel D (2000). Social support and salivary cortisol in women with metastatic breast cancer. Psychosom Med.

